# Systematic assessment of microRNAs associated with lung cancer and physical exercise

**DOI:** 10.3389/fonc.2022.917667

**Published:** 2022-08-30

**Authors:** Yang Liu, Libo He, Wang Wang

**Affiliations:** ^1^ Department of Central Laboratory, The First People’s Hospital of Huzhou, First Affiliated Hospital of Huzhou University, Huzhou, China; ^2^ Key Laboratory of Bio-Resource and Eco-Environment of Ministry of Education, College of Life Sciences, Sichuan University, Chengdu, China; ^3^ Department of Medicine, Nanchang Medical College, Nanchang, China

**Keywords:** microRNA, lung cancer, physical exercise, bioinformatics, prognosis

## Abstract

It has long been evident that physical exercise reduces the risk of cancer and improves treatment efficacy in tumor patients, particularly in lung cancer (LC). Several molecular mechanisms have been reported, but the mechanisms related to microRNAs (miRNAs) are not well understood. MiRNAs modulated various basic biological processes by negatively regulating gene expression and can be transmitted between cells as signaling molecules. Recent studies have shown that miRNAs are actively released into the circulation during exercise, and are deeply involved in cancer pathology. Hence, the role of exercise intervention in LC treatment may be further understood by identifying miRNAs associated with LC and physical activity. Here, miRNAs expression datasets related to LC and exercise were collected to screen altered miRNAs. Further bioinformatic approaches were performed to analyze the value of the selected miRNAs. The results identified 42 marker miRNAs in LC, of which three core-miRNAs (has-miR-195, has-miR-26b, and has-miR-126) were co-regulated by exercise and cancer, mainly involved in cell cycle and immunity. Our study supports the idea that using exercise intervention as adjuvant therapy for LC patients. These core-miRNAs, which are down-regulated in cancer but elevated by exercise, may act as suppressors in LC and serve as non-invasive biomarkers for cancer prevention.

## Introduction

Lung cancer (LC) is a heterogeneous disease, including small cell lung cancer and non-small cell lung cancer (NSCLC). NSCLC comprises 85% of new LC cases and contains two major histological types, lung adenocarcinoma (LUAD) and lung squamous cell carcinoma (LUSC), of which LUAD is the most common subtype (1, 2). Albeit the advancement in therapeutic methods and detection tools, LC was still the leading cause of cancer-related death, with an estimated that LC patients will rise to ten million by 2030 (3). Long-term morbidity and serious complications not only bring pain and suffering to patients but also impose a huge social and economic burden on society. Currently, except the standard treatments such as chemotherapy, radiotherapy, surgical resection and recently immunotherapy, several adjuvant therapies have been applied in LC patients to enhance the efficacy of treatments and improve quality of life (4–6). Adjuvant therapies mainly include nutrition optimization, dietary customization, and exercise therapy (7, 8). For patients with metabolic disorders, hormonal imbalance, diabetes mellitus, unhealthy lifestyle, aging, adjuvant therapies have been shown beneficial in reducing cancer risk and improving patient prognosis (9–11). Among these adjuvant therapies, exercise interventions have shown to be associated with inhibiting deterioration and decreasing mortality in multiple cancers, especially lung cancer (12–16).

Physical exercise could provide numerous benefits for cancer patients, such as strengthening lung function, improving metabolism, enhancing immunity, relieving pain and depressive symptoms (15, 17). And several studies have shown that exercise intervention can reduce the symptoms of dyspnea, coughing, anxiety and suppress tumor growth in LC (18–21). But the torture of tumors and the pain of treatment cause adverse physical and psychological effects on LC patients, leading to higher psychological distress, resulting in a reduction of physical activity, and forming a vicious cycle (22–24). Therefore, exercise interventions become more necessary for LC patients and are frequently applied in coordination with other treatments. Recently studies have also shown that regular exercise for LC patients could improve cardiorespiratory fitness, reduce pulmonary complications and shorten postoperative hospitalization (25–28).

All this evidence prompted researchers to study the effects of physical exercise on LC. Earlier studies showed that exercise can modulate epigenetic modifications, namely DNA methylation and post-translational histone modification, which in turn intervene in metabolism, biosynthesis and development, as well as redox signaling, DNA repairing, and aging (29–32). While recent researches indicate that non-coding RNA, especially the circulating miRNA, is closely associated with physical exercise (33). Circulating miRNAs responsive to exercise in brain, muscle, kidney and lung, serve as physiological mediators of exercise-induced adaptation (34). MiRNAs in cancer exosomes transfer to target cells for communication, act as initiators for pre-metastatic niche formation (35–37). Therefore, it is necessary to study physical exercise and LC-regulated miRNAs to assess the therapeutic potential of exercise intervention. However, convincing data on this topic is still insufficient. In this study, we collected miRNA expression datasets from Gene Expression Omnibus (GEO) database and screened out three core-miRNAs that were regulated by exercise and associated with cancer pathology. Further bioinformatics analysis found that these core-miRNAs were associated with a better prognosis of LC.

## Materials and methods

### Data collection and processing

The miRNA and mRNA expression profiles of LC and exercise were obtained from National Center for Biotechnology Information (NCBI) Gene Expression Omnibus (GEO) database (https://www.ncbi.nlm.nih.gov/geo/). The search terms we used included “lung cancer”, “exercise” and “Homo sapiens”. Discarded datasets obtained from animals, cell lines, or other without normal control. And the corresponding pan-cancer expression profiles and clinical data were obtained from The Cancer Genome Alters (TCGA) database (https://portal.gdc.cancer.gov/). **Tables S1** and **S2** show the information from our collected datasets. The differential genes were analyzed using the GEO2R online tool (https://www.ncbi.nlm.nih.gov/geo/geo2r/), which was based on R language.

### Prediction of potential target genes of miRNA

The target genes of miRNA were predicted using ENCORI (https://starbase.sysu.edu.cn/index.php), which is an open-source platform for studying the miRNA-mRNA interaction through seven algorithms (PITA, TNA22, miRmap, microT, miRanda, PicTar, TargetScan). The screened miRNAs were typed into this platform, and targeted mRNAs produced by at least four prediction algorithms were considered. To validate these predictions, the candidate target genes were compared with the differential genes in five LC datasets. And the miRNA-mRNA network was visualized by Cytoscape 3.9.1.

### Functional enrichment analysis

Various tools were used to analyze the function of our selected miRNAs and mRNAs. Including miRNA Enrichment Analysis and Annotation tool (miEAA, https://ccb-compute2.cs.uni-saarland.de/mieaa2/), RNALocate (https://www.rna-society.org/rnalocate/), KEGG Orthology Based Annotation System (KOBAS 3.0, http://kobas.cbi.pku.edu.cn/home.do), DAVID Bioinformatics Resources (https://david.ncifcrf.gov/home.jsp), and LinkedOmics (http://www.linkedomics.org/login.php). The miEAA was used for functional enrichment analysis of miRNA sets. Subcellular localization analysis of miRNAs was performed by RNALocate. For the genes, KOBAS and DAVID were used to perform GO categories, KEGG pathway, and Reactome pathway analysis. The LinkedOmics dataset was used for GSEA function enrichment analysis of gene sets in lung cancer. R drawing package ggplot2 was utilized for making bubble chart, bar graph and heatmap.

### Exploration of signatures associated with cancer

Gene expression and cancer pathway activity in LUAD and LUSC were analyzed by a web-based platform for Gene Set Cancer Analysis (GSCALite, http://bioinfo.life.hust.edu.cn/web/GSCALite/). Tumor-infiltrating immune cells analysis was performed by TIMER (http://cistrome.dfci.harvard.edu/TIMER/). Survival analysis based on the expression status of mRNA/miRNA was performed by GEPIA 2.0 (http://gepia.cancer-pku.cn/) or R statistical package software survminer (version 0.4.9) and survival (version 3.2-10). Correlations between the gene sets of interest and functional states were performed by Cancer Single-cell state Atlas (CancerSEA, http://biocc.hrbmu.edu.cn/CancerSEA/). Tumor stromal score was analyzed by R statistical package software estimate (version 2.0.0), which was based on the gene expression profiles retrieved from TCGA. Tumor stemness scores were calculated by one-class logistic regression algorithm, and Spearman correlation analysis based on mRNA (RNAss) and DNA-methylation (DNAss) datas of TCGA pan-cancer samples were downloaded from Xena browser (https://xenabrowser.net/datapages/).

### Statistical analysis

The log2FC values of miRNAs/mRNAs were normalized by GEO2R. For LC datasets, miRNAs with *p*-value < 0.05 were selected. For mRNAs and exercise datasets, *p*-value < 0.01 were selected. The Kaplan–Meier method was used to generate overall survival curves, and log-rank *p*-value < 0.05 were selected. The association between gene expression, stromal score and stemness score was tested with Spearman correlation.

## Result

### Identification of LC-associated miRNAs

**Figure 1:**
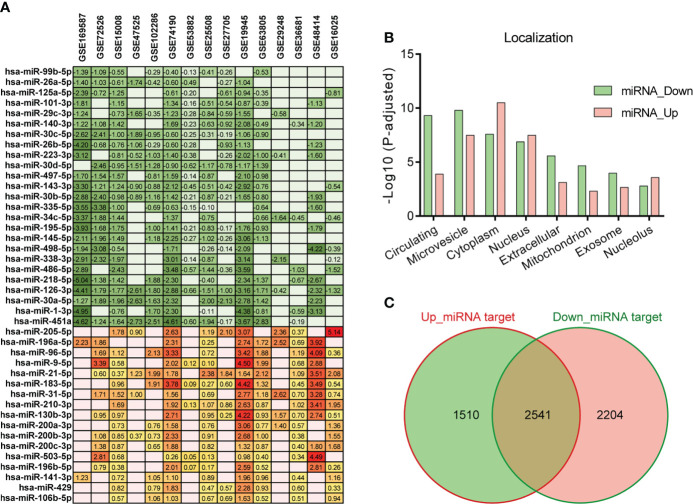
Differentially expressed miRNAs in LC. **(A)** Differentially expressed miRNAs (*p* < 0.05) in tumor compared to normal in at least nine out of fifteen LC datasets. The number represents the value of log2FC determined by GEO2R analysis, and the red/green scale boxes represent the up- or down-regulated miRNAs. **(B)** Subcellular localization of the selected miRNAs was obtained by RNALocate. **(C)** Target genes of the up- and down-regulated miRNAs were predicted by ENCORI. The overlapping parts were deleted from the predicted gene lists and only considered the two flanking genes.

The miRNA expression profiles related to LC were obtained from GEO DataSets. Excluding the datasets obtained from circulating fluids, or samples without normal control, or animal models, or *in vitro*, we selected 15 datasets of LC (**Table S1**). These datasets were used to comprehensively evaluate the LC-associated miRNAs. Differentially expressed miRNAs between tumor and normal were analyzed by GEO2R. Considering the different sample collection methods among datasets and the complex pathological features of patients, a stringent screening strategy may omit important miRNAs, so we adopted a relatively loose threshold (*p* < 0.05) (38–40). As a result, a total of 42 dysregulated miRNAs were found in at least nine out of fifteen selected datasets. Of these miRNAs, 25 were down-regulated and 17 were up-regulated in tumor samples compared to normal (**Figure 1A**). All of the down-regulated miRNAs were identified as circulating miRNAs by RNALocate database, of which 23 were mainly located in microvesicles. And the up-regulated miRNAs were primarily localized in cytoplasm (**Figure 1B**). In addition, excluding the overlapping parts, we predicted 2204 target genes of down-regulated miRNAs and 1510 target genes of up-regulated miRNAs using ENCORI database (**Figure 1C**, **Table S3**).

### Functional enrichment analysis of LC-associated miRNA target genes

**Figure 2:**
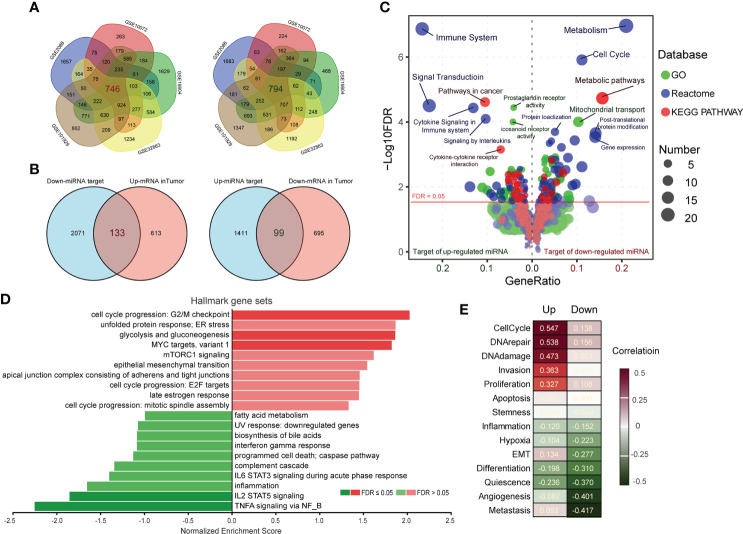
Functional enrichment of target genes of miRNAs. **(A)** Up- and down-regulated genes (*p* < 0.01) in tumors compared to normal were obtained by GEO2R analysis, and the overlapping parts of the five LC datasets were selected as candidate target genes of miRNAs. **(B)** Comparing the miRNA target genes list predicted by ENCORI with the merged dysregulated genes list of five LC datasets, the overlapping parts were considered to be the targets of miRNAs. **(C)** The GO (green), Reactome (blue) and KEGG pathway (red) enrichment analysis of miRNA target genes was performed by KOBAS 3.0. The size of the knots represents the number of enriched genes. **(D)** The target genes were ranked by the mean of log2FC in the five LC datasets, and were annotated by Gene Set Enrichment Analysis (GSEA) according to Hallmark gene sets of cancer. **(E)** Functional states of target genes in LC were analyzed by CancerSEA, and the red/green scale boxes represent up- or down-regulation.

To further corroborate the target prediction results of LC-associated miRNAs, we separately merged the up-and down-regulated differential genes (*p* < 0.01) from five LC datasets containing normal controls (**Figure 2A**, **Table S2**), and compared them with the prediction results. Considering the anti-correlation between miRNA and its targets, we further screened 133 target genes of down-regulated miRNAs and 99 target genes of up-regulated miRNAs (**Figure 2B**, **Table S4**). Gene ontology (GO), Reactome and KEGG pathway analysis were performed to identify the cellular processes associated with target genes of LC-associated miRNAs. The results showed that plenty of cellular processes were modulated by these genes (**Figure 2C**). For target genes of down-regulated miRNA, “Metabolism” and “Cell Cycle” in Reactome, “Metabolic pathways” in KEGG, and “Mitochondrial transport” in GO were the highest enrichment. For target genes of up-regulated miRNA, “Immune System” in Reactome, “Pathways in cancer” in KEGG, and “prostaglandin receptor activity” in GO were the highest enrichment. All of these processes were known to be associated with the development and progression of cancer. Ranked these genes according to the average value of Log2FC in five LC datasets, and subjected to Gene Set Enrichment Analysis (GSEA) according to cancer hallmark gene sets. It was found that “cell cycle progression” and “TNFA signaling *via* NFκB” were the most enriched terms (**Figure 2D**). And functional states of single-cell analyzed by CancerSEA also showed that “CellCycle” and “Metastasis” were the most relevant terms (**Figure 2E**). Overall, these target genes of selected miRNAs have closely linked with LC development, especially the target genes of which down-regulated miRNAs are significantly associated with the cell cycle.

### Interaction analysis of exercise- and LC-associated miRNAs

**Figure 3:**
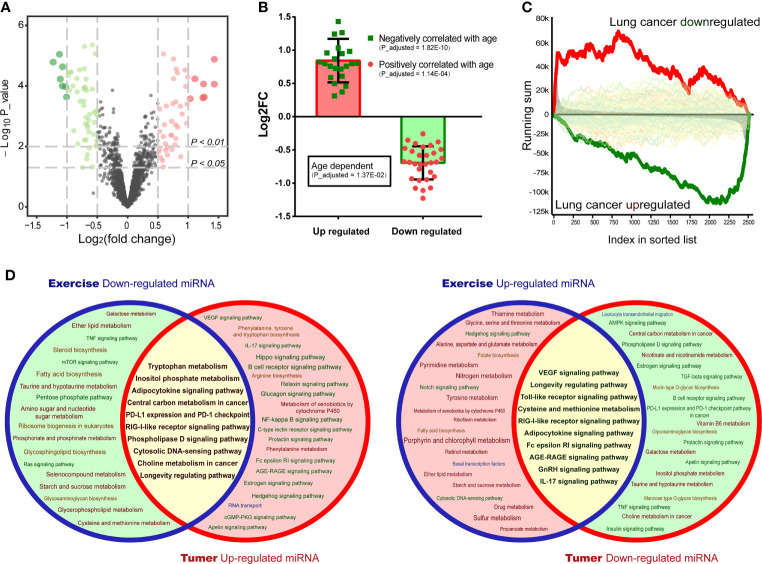
Interaction analysis of exercise/LC-associated miRNAs. **(A)** Volcano plots draw the regulated circulating miRNAs in plasma after exercise in a dataset of GSE133910. **(B)** Age-dependent genes were annotated by miEAA, and each knot represents an age-related differentially expressed miRNA (*p* < 0.01) in the exercise dataset. **(C)** The circulating miRNAs were ranked by the value of log2FC, and disease correlation analysis was performed by GSEA. The enriched(red) or depleted(green) subcategory was shown based on the running sum. **(D)** Pathway prediction analysis of exercise-regulated miRNAs (*p* < 0.01) and screened LC miRNAs was performed by miEAA. The overlapping regions (yellow) represent the co-intervention part.

The positive effects of healthy physical activity on LC prevention and prognosis have been characterized. However, the understanding of the underlying mechanisms is still limited. It was hypothesized that exercise-induced modulation of circulating miRNA may be associated with LC development. To corroborate this hypothesis, a differential analysis between circulating miRNAs after and before exercise was performed (**Figure 3A**). The differential miRNAs (*p*<0.01) were annotated by miEAA. It was found that up-regulated circulating miRNAs were negatively correlated with age, while the down-regulated were positively correlated with age (**Figure 3B**). And gene set enrichment analysis revealed that these circulating miRNAs were associated with LC-driven gene. The up-regulated circulating miRNAs were enriched in LC down-regulated gene sets, while the down-regulated were enriched in LC up-regulated gene sets (**Figure 3C**). These results suggested that exercise may have anti-aging effects and benefit the health of LC patients through miRNA modulation. Hence, we compared the functional annotation results of exercise-down-regulated miRNAs and LC-up-regulated miRNAs, as well as exercise-up-regulated miRNAs and LC-down-regulated miRNAs (**Figure 3D**). Found that many important cancer-related pathways (such as “Central carbon metabolism in cancer”, “PL-L1 expression and PD-1 checkpoint” and “RIG-I-like receptor signaling pathway”, as well as “VEGF signaling pathway”, “Toll-like receptor signaling pathway” and “Cysteine and methionine metabolism”) were intervened by the exercise-regulated miRNAs, especially the immune- and metabolism-related pathways (**Figure 3D**). Therefore, these results suggest that exercise interventions may be a valuable strategy to prevent or limit LC risk.

### Co-target genes of exercise- and LC-associated miRNAs affected immune infiltration and cell cycle

**Figure 4:**
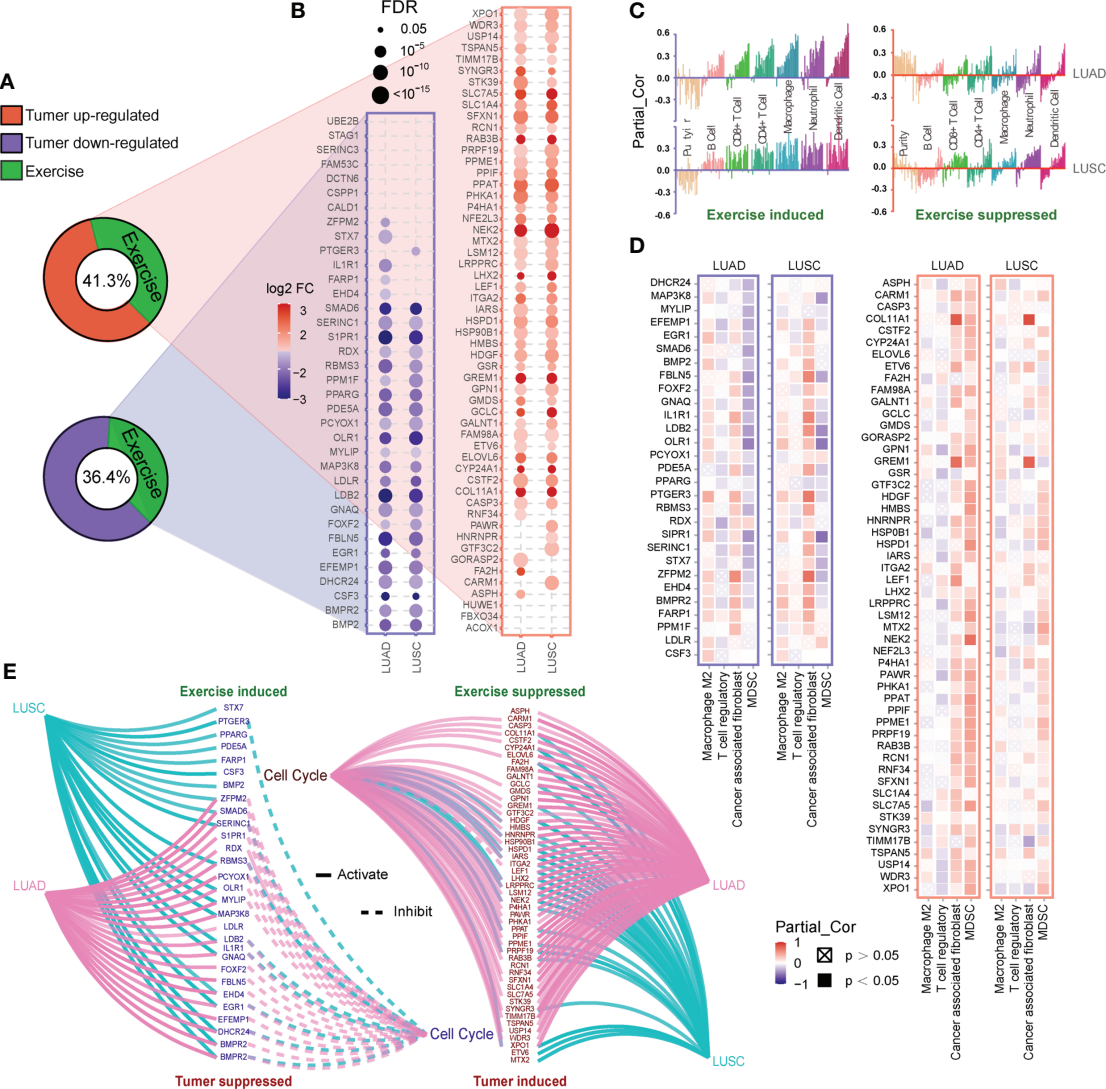
Functional analysis of co-target genes. **(A, B)** Based on the revers analysis function of RNCORI, 41.3% up-regulated and 36.4% down-regulated target genes of LC-associated miRNA **(A)** could be inversely regulated by exercise-associated miRNAs (green). And these co-target genes **(B)** were verified by the mRNA expression module of GCSALite based on TCGA expression data (LUAD and LUSC). **(C, D)** The correlations between co-target genes and infiltration of six immune cell types **(C)** and four immunosuppressive cell types **(D)** in LUAD and LUSC were shown. Every bar in **(C)** represents a co-target gene. The correlations were depicted by purity-corrected partial Spearman’s rho values (Partial_Cor) through TIMER 2.0. **(E)** The activity of co-target genes in cancer-related cell cycle pathway was defined and presented by the pathway activity module of GCSALite.

Considering the positive role of exercise, we obtained co-target genes by comparing the targets of exercise and LC-associated miRNAs. 55 of 133 (41.3%) up-regulated co-target genes and 36 of 99 (36.4%) down-regulated co-target genes in LC were inversely regulated by exercise-associated miRNAs (**Figure 4A**). The expression of these genes was further verified in The Cancer Genome Atlas (TCGA) LUAD/LUSC cohort, and 52 up-regulated and 29 down-regulated co-target genes in LC were validated (**Figure 4B**). Then, we explored the correlation of the validated co-target genes with immune infiltration by TIMER database. The down-regulated genes in LC (exercise-induced genes) showed significant positive correlations of six immune cell types (B cell, CD8+ T cell, CD4+ T cell, Macrophage, Neutrophil and Dendritic cell) (**Figure 4C**). We also assessed the correlation of these genes with four immunosuppressive cells (Macrophage M2, T cell regulatory, Cancer-associated fibroblast, and Myeloid-derived suppressor cells) which were known to promote T cell exclusion. It was found that exercise-induced genes were negatively correlated with Myeloid-derived suppressor cells (MDSC), while the up-regulated genes in LC (exercise-suppressed genes) were positively correlated with MDSC, especially in LUAD (**Figure 4D**). Since aberrant cell cycle activity is a feature of many cancers (41), cell cycle annotation analysis was performed by GSCALite. We found that almost all the exercise-suppressed genes were involved in LC cell cycle activation, whereas the exercise-induced genes do the opposite (**Figure 4E**). These results demonstrated that exercise may modulate immune infiltration and inhibit cancer cell proliferation by regulating target genes of LC-associated miRNA.

### Core-miRNAs regulated by exercise and LC are associated with patient prognosis

**Figure 5:**
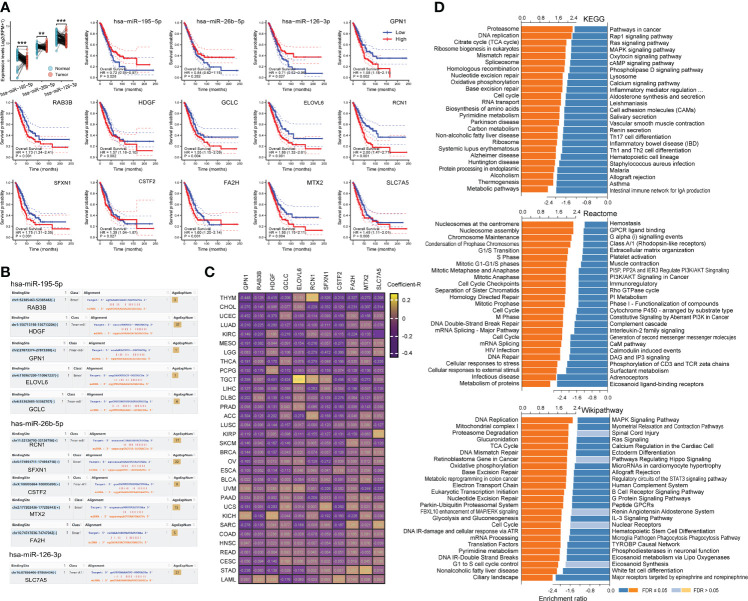
Binding site of core-miRNAs and their effects. **(A)** The expression of core-miRNAs in paired LC samples in TCGA was shown, and the effects of miRNA/mRNA on the overall survival of LUAD patients were calculated by Kaplan-Meier method. **(B)** The binding sites of miRNAs were shown. **(C)** Heatmap shown the expression correlation Coefficient-R of miRNA-mRNA calculated by ENCORI across pan-cancer. **(D)** The functional enrichment analysis of core-miRNA target genes in LUAD was performed by GSEA through the LinkCompare module of LinkedOmics.

We explored the association of co-target genes with LC prognosis (**Figure S1A**). Results showed that LUAD was more susceptible to the co-target genes, especially the 52 genes suppressed by exercise. And these genes were significantly associated with poor prognosis of LUAD (log-rank *p* = 0.0011, hazard ratio = 1.6), while weakly associated with LUSC (**Figure S1A**). Pathway analysis found that these genes were mainly related to cell metabolism (**Table S5**). We also constructed a miRNA-mRNA network (**Figure S1B**), showed that there were three core-miRNAs (hsa-miR-195-5p, hsa-miR-26b-5p and hsa-miR-126-3p) were co-regulated by exercise and LC (**Figure S1B**). Of these genes, 29 (56%) were modulated by core-miRNAs and were associated with high hazard ratios across multiple LC datasets (**Figure S1C**). By detecting the TCGA lung cancer cohort, core-miRNAs expression was higher in paired normal tissues and associated with a better prognosis (**Figure 5A**). Among core-miRNAs targe genes, eleven hub-genes expressions were significantly associated with LUAD prognosis (log-rank *p* < 0.05), and overexpression indicated poor prognosis (**Figure 5A**). The binding sites of core-miRNAs were shown (**Figure 5B**), and the expression of hub-genes were inversely correlated with core-miRNAs in nearly all TCGA cancer types (**Figure 5C**). Combing GSEA enrichment results from KEGG, Reactome and Wikipathway databases, shown that hub-genes were positively correlated with cell replication pathway (such as “DNA replication” in KEGG and Wikipathway, “Nucleosomes at the centromere” in Reactome), and inversely correlated with many immune relevant terms (**Figure 5D**). These results suggested that core-miRNAs were associated with exercise intervention in LC progression, and the oncogenic role of these miRNAs may be achieved through regulating cell replication and immunity signaling.

### Expression and function of hub-genes in pan-cancer

**Figure 6:**
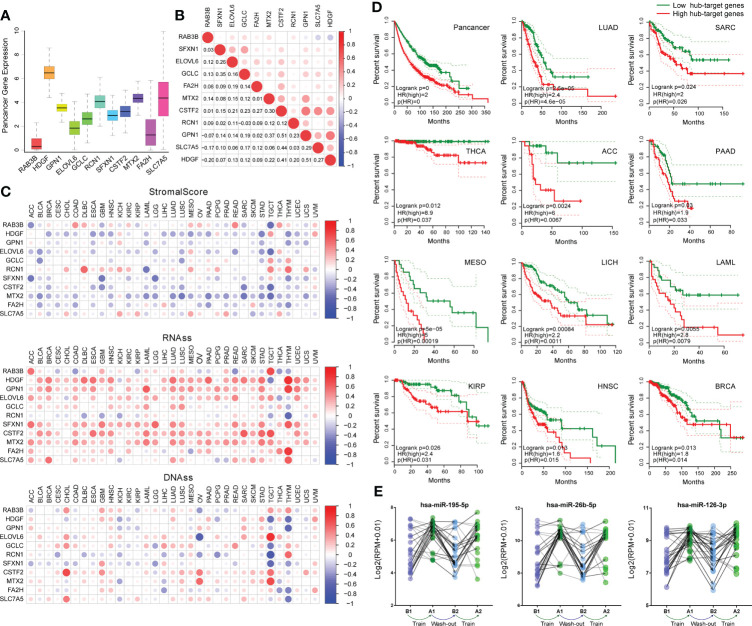
Correlation of hub-genes with cancer pathology and prognosis. **(A)** Boxplot shown the expression of hub-genes across pan-cancer in TCGA. **(B)** Correlation plot based on Spearman test shown the relationship of hub-genes expression across pan-cancer. **(C)** Correlation matrix plots shown the association between hub-genes expression and stromal scores (based on ESTIMATE algorithm), as well as cancer stemness scores (RNAss: based on RNA expression, DNAss: based on DNA methylation). The size of the dots represents the value of Spearman test, and the red/blue dots indicate a positive/negative correlation. **(D)** Kaplan-Meier plots of hub-genes showed differential overall survival outcomes (*p* <0.05) across pan-cancer. **(E)** The expression of core-miRNAs in an exercise dataset (GSE133910). At four time points (B1, A1, B2 and A2) plasma samples were collected. Volunteers completed eight weeks of training, followed by a wash-out phase (eight weeks), and another eight weeks of training analogous to the first phase.

Utilizing the pan-cancer atlas project of TCGA, we comprehensively analyzed the expression and function of hub-genes regulated by core-miRNA at pan-cancer level through high-throughput gene expression data and clinical information. The expression of hub-genes was shown (**Figure 6A**), and the correlation coefficient between every two of them was calculated (**Figure 6B**). Most of the coefficients are positive, suggesting that these genes have synergistic effects. Further research has shown that the expression of most hub-genes was negatively correlated with the stromal score calculated by ESTIMATE algorithm, suggesting that tumors with high hub-genes expression contained lower immune cells and higher cancer cells (**Figure 6C**). While stemness score (RNAss and DNAss) was positively correlated with most hub-genes, especially RNAss, suggesting that higher hub-genes expression is associated with stronger activity of cancer stem cells and tumorigenesis (**Figure 6C**). Survival curve analysis was performed (**Figure 6D**), shown that high expression of hub-genes was linked to poor prognosis in pan-cancer, especially in LUAD, SARC, THCA, ACC, PAAD, MESO, LICH, LAML, KIRP, HNSC and BRCA (log-rank *p* < 0.05). In addition, these three core-miRNAs were up-regulated in plasma after 8 weeks of training (A1), returned to basal state (B2) after 8 weeks off, and up-regulated again after retraining (A2) (**Figure 6E**). Overall, these results indicated that regular physical exercise may associate with cancer prevention and reflect a better prognosis.

## Discussion

It has been demonstrated that lifestyle plays important role in maintaining health and preventing disease. Regular physical exercise is a flexible, inexpensive, and most important, effective way to keep healthy. It has been used as an adjuvant therapeutic strategy for much of chronic disease treatment, such as obesity, diabetes, osteoporosis and angiocardiopathy (42, 43). In cancers, growing studies have shown that physical exercise is associated with lower cancer risk. For athletes, the overall cancer incidence was low, LC risk decreased most (44, 45). 2.5 to 5 hours of moderate-intensity physical activity per week for adults was recommended by US Physical Activity Guidelines Advisory Committee, which has been shown to be beneficial for cancer prevention (46). Compared with sedentary lifestyle, physical activity individuals’ LC risk significantly decreased (12–15, 47). And physical inactivity in LC is often associated with poor prognosis and increased recurrence risk (13, 48). Despite extensive studies conducted on exercise interventions, the underlying mechanisms have not been elucidated yet. Recently, miRNAs have emerged as biomarkers, since numerous mature miRNAs enter the circulation *via* extracellular vesicles during various diseases (49). For cancer cells, circulating miRNAs are required to regulate microenvironments, initiate metastasis and suppress immune responses, and serve as diagnostic/prognostic markers (37, 50). Previous studies have also shown that exercise could modulate circulating miRNAs by regulating the release of extracellular vesicles (33, 51). Therefore, it is valuable to use circulating miRNA as an entry point for understanding the interaction between exercise and LC.

MiRNAs associate with argonaute proteins to form a miRNA-induced silencing complex (RISC). RISC inhibits the translation of genes into proteins by directly binding the complementary sequences located in the untranslated regions of mRNA. For cancers, miRNAs could silence tumor suppressors or stimulate oncogene expression to interfere with cancer progression (35, 36, 50, 52). While, several studies have demonstrated that global miRNA expression was usually reduced in cancers since some miRNAs act as negative regulators of genes to block carcinogenesis, and tumors need to unlock these control genes to obtain the ability of sustaining proliferative and avoiding immune destruction (53, 54). In this context, the integrated analysis of fifteen different miRNA expression profiling datasets was performed and identified a set of 42 dysregulated miRNAs in LC, including 25 down-regulated (**Figure 1**). And plenty of cellular processes were found to be intervened by these dysregulated miRNAs *via* combining the predicted targets of miRNAs with the differential expressed gene in LC RNAseq datasets (**Figure 2**). Cell cycle and metabolism were mainly associated with down-regulated miRNAs, immunity and inflammation were associated with up-regulated miRNAs. Additionally, some of these miRNAs have already been detected as circulating miRNAs in plasma of cancer patients and proposed as potential biomarkers for various cancer types, such as has-mir-195 (55), has-mir-26b (56), has-mir-126 (57), hsa-mir-29c (58), hsa-mir-125a (59), hsa-mir-223 (60), has-mir-210 (61) and has-mir-141 (62).

Circulating miRNAs, attached to proteins or loaded in extracellular vesicles, are present in various body fluids. It can be actively secreted into the extracellular space as signaling molecules by cells under external stimulation (63). Recent studies have shown that exercise of different intensities and durations can induce a massive release of extracellular vesicles into the circulation to modulate individual biological processes (33, 51). However, the ability of physical exercise to modulate miRNAs relevant to LC prevention has not been well investigated yet. In the present study, we proposed integrated analysis of exercise-regulated circulating miRNAs with 42 miRNAs we screened in LC datasets (**Figure 3**). This approach revealed that some biological processes of cancer development tend to be relocked by exercise intervention *via* promoting the secretion of circulating miRNAs, such as VEGF signaling pathway, toll-like receptor, cysteine and methionine metabolism, and IL-17 pathway. Conversely, the functions of exercise-downregulated miRNAs were complex and may be involved in cancer defense *via* cytosolic DNA-sensing pathway. The DNA-sensing mechanism is the molecular basis to produce immune response (64). Further analysis also found six immune cells were positively associated with exercise intervention (**Figure 4C**). With miRNA target prediction, we found that most target genes of LC-associated miRNAs can be regulated by exercise. The genes silenced by exercise up-regulated miRNAs were associated with cell cycle activation and promoted tumor infiltration of MDSC in LUAD. While for the down-regulated miRNAs in exercise, the effect was opposite (**Figure 4**). These analyses confirmed that exercise is strongly involved in LC pathology, especially in cell cycle and immune infiltration pathways.

During the above process, we identified three core-miRNAs (has-miR-195, has-miR-26b and has-miR-126), which were repressed in LC but elevated by exercise. These core-miRNAs circulating in plasma were also found to be increased in different acute or chronic training projects, including cycling, swimming and marathon running (65–71). In a weight-loss trial, the expression of core-miRNAs was much higher in the slimming success cohort (72). And plenty of evidence has confirmed that these miRNAs were able to transmit between cells (73). Our results showed that core-miRNAs and their targets have a strong involvement in DNA replication, immune system and prognosis of patients in LC (**Figure 5**). And several studies have already highlighted their anticancer effect. For example, the increased level of has-miR-195 is associated with inhibition of proliferation and angiogenesis, and enhances T cell activation in several cancer types including LC (74–76). Similarly, has-miR-26b has a strong involvement in cell cycle transition, apoptosis induction, and improve chemosensitivity (77–79). Furthermore, has-miR-126 expression also could reduce tumor growth, inhibit metastasis, and contribute to enhancing cisplatin cytotoxicity (80–82). For the targets of the core-miRNAs, we identified eleven hub-genes associated with overall survival, all of which high expression indicated poor prognoses. Therefore, these core-miRNAs we identified may play critical roles in exercise intervention against LC progression. Additionally, we analyzed the correlation between hub-genes expression and tumor stem cell score across 33 cancer types. The results showed that high expression of hub-genes in pan-cancer, including LC, represented lower stromal cell content and higher stem cell characteristics (**Figure 6**). Suggesting these hub-genes play a role in maintaining cancer stem cells and act as tumor promoters. High express levels of these hub-genes also represent a high risk in pan-cancer, especially in LUAD, SARC, THCA, ACC, PAAD, MESO, LICH, LAML, KIRP, HNSC and BRCA.

In conclusion, the above bioinformatics analysis we performed allows us to identify specific miRNAs to predict the risk of LC, and imply the connection between physical exercise and cancer prevention. Regular exercise can alter circulating miRNAs in plasma, which act as signaling molecules transmitted between cells and may regulate tumor physiology and delay the progression of cancer by modulating cell cycle and immune system. Supports the idea of prescribing physical exercise as adjuvant therapy for LC patients, especially for those who are sedentary, depressed and anxious. Of note, after a period of cessation of exercise, the elevated miRNAs returned to their base state and increased again after resumption. This reminds us that the benefits of physical exercise require unremitting efforts to manifest, and the same should be true for any other adjuvant therapies.

## Data availability statement

Publicly available datasets were analyzed in this study. This data can be found here: https://www.ncbi.nlm.nih.gov/geo/.

## Author contributions

YL, LH, and WW contributed equally to this work. All authors contributed to the article and approved the submitted version.

## Conflict of interest

The authors declare that the research was conducted in the absence of any commercial or financial relationships that could be construed as a potential conflict of interest.

## Publisher’s note

All claims expressed in this article are solely those of the authors and do not necessarily represent those of their affiliated organizations, or those of the publisher, the editors and the reviewers. Any product that may be evaluated in this article, or claim that may be made by its manufacturer, is not guaranteed or endorsed by the publisher.
