# Supplementary material for: Systematic assessment of microRNAs associated with lung cancer and physical exercise
Source: Front Oncol. 2022 Aug 30;12:917667. doi: 10.3389/fonc.2022.917667 (PMC9468783; doi:10.3389/fonc.2022.917667)
Supplement: Supplementary file 2 [file Image_1.pdf]

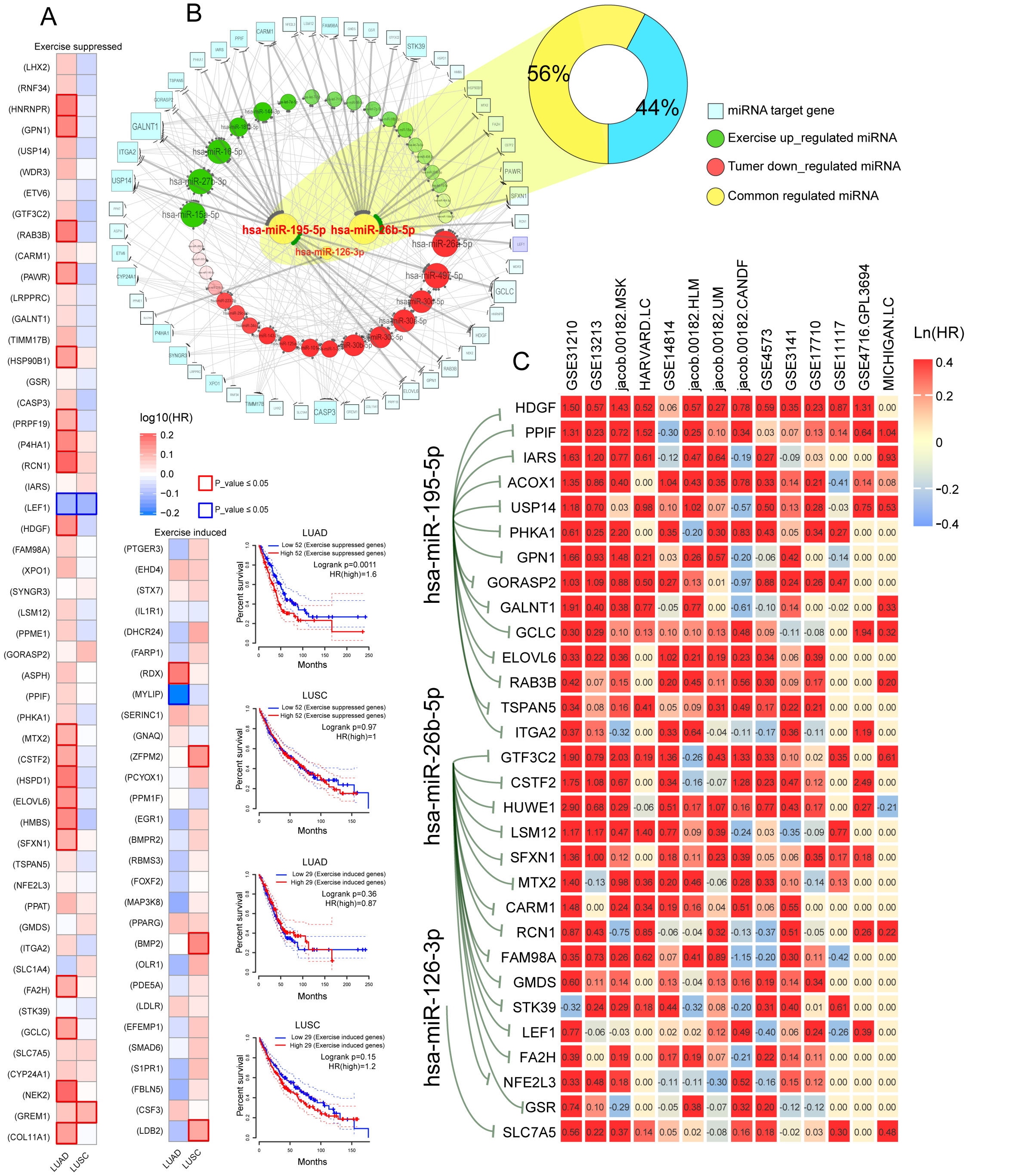

Figure S1 Correlation of co-target genes with LC prognosis.

(A) Survival heat map of co-target genes was constructed by Mantel-Cox test module of GEPIA 2.0 in LUAD/LUSC. “Exercise suppressed” represents the target genes of up-regulated miRNAs after exercise, which are up-regulated in LC. “Exercise induced” represents the target genes of down-regulated miRNAs after exercise, which are down-regulated in LC. Kaplan-Meier plots of co-target genes shown overall survival outcomes in LUAD/LUSC, and Log-rank p ＜ 0.05 was considered to be significant. (B) The interaction of up-regulated miRNAs after exercise and down-regulated miRNAs in LC with co-binding targets. The overlapping part (yellow) indicated that miRNAs were co-regulated by exercise and LC (core-miRNAs), and target genes of core-miRNAs accounted for 58% of the total. (C) Heat map shown natural logarithmic hazard ratios (Ln (HR)) for core-miRNA targe genes calculated by PrognoScan across multiple LC datasets.
